# Supplementary figures and images for: Practical fluorescence reconstruction microscopy for large samples and low-magnification imaging
Source: PLoS Comput Biol. 2020 Dec 23;16(12):e1008443. doi: 10.1371/journal.pcbi.1008443 (PMC7802935; doi:10.1371/journal.pcbi.1008443)

## PCC vs. Modified $P$

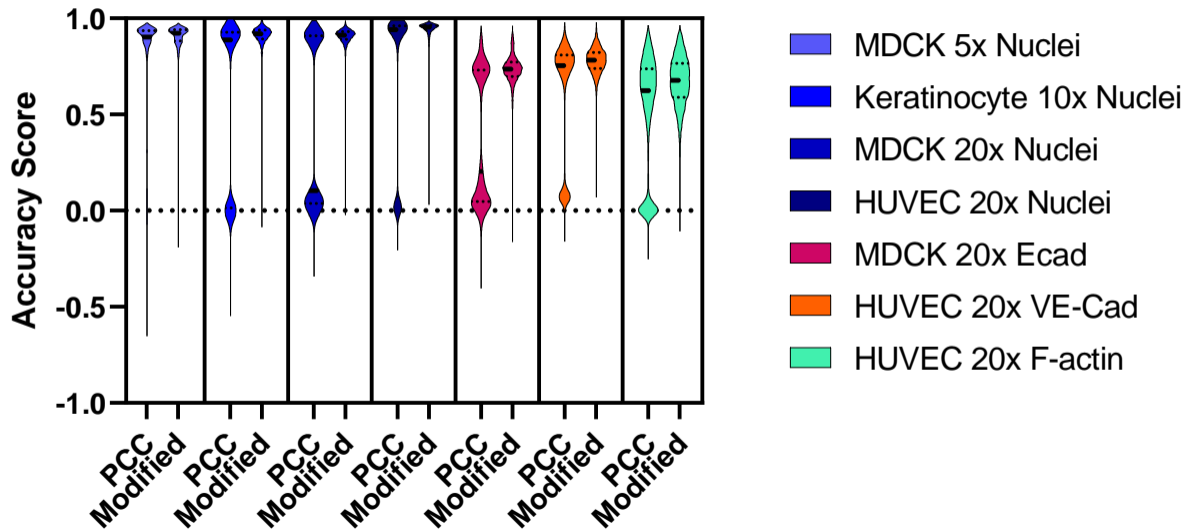

Supplement: S1 Fig — To ensure a fairer comparison, outliers were not removed; only intensity thresholding was performed to produce the modified P from the PCC. By filtering the PCC results by an intensity threshold in the fluorescent images, we remove low-scoring background images, which bias our accuracy score on the complete dataset. Visual inspection of the plot reveals the low-scoring images as “bumps” near 0.0. S1 Table summarizes the statistics. (PDF) [file pcbi.1008443.s001.pdf]

## Network Depth and Accuracy

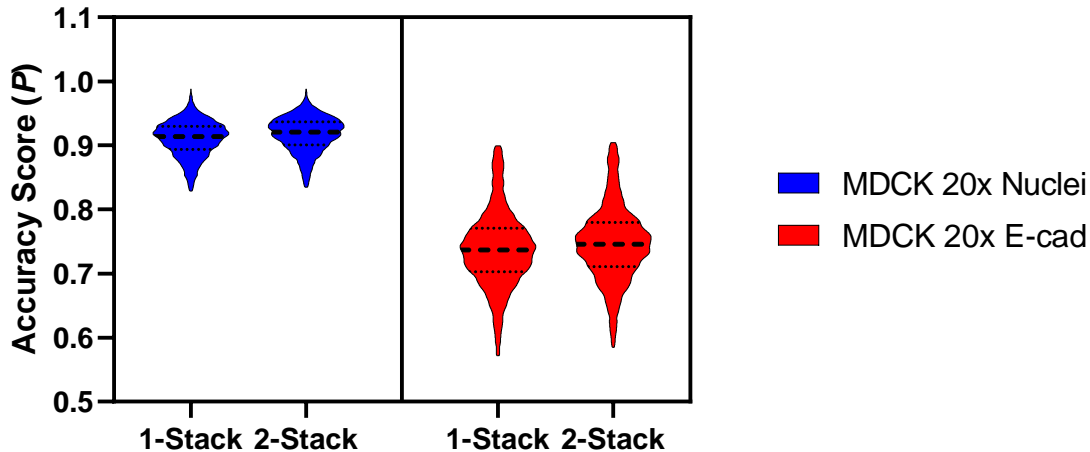

Supplement: S4 Fig — Training conditions were otherwise unchanged. Accuracy scores, as reported in terms of the modified P (see Methods were comparable. N = 10,000 sub-image patches for each test condition. (PDF) [file pcbi.1008443.s004.pdf]

## Data Augmentation Results

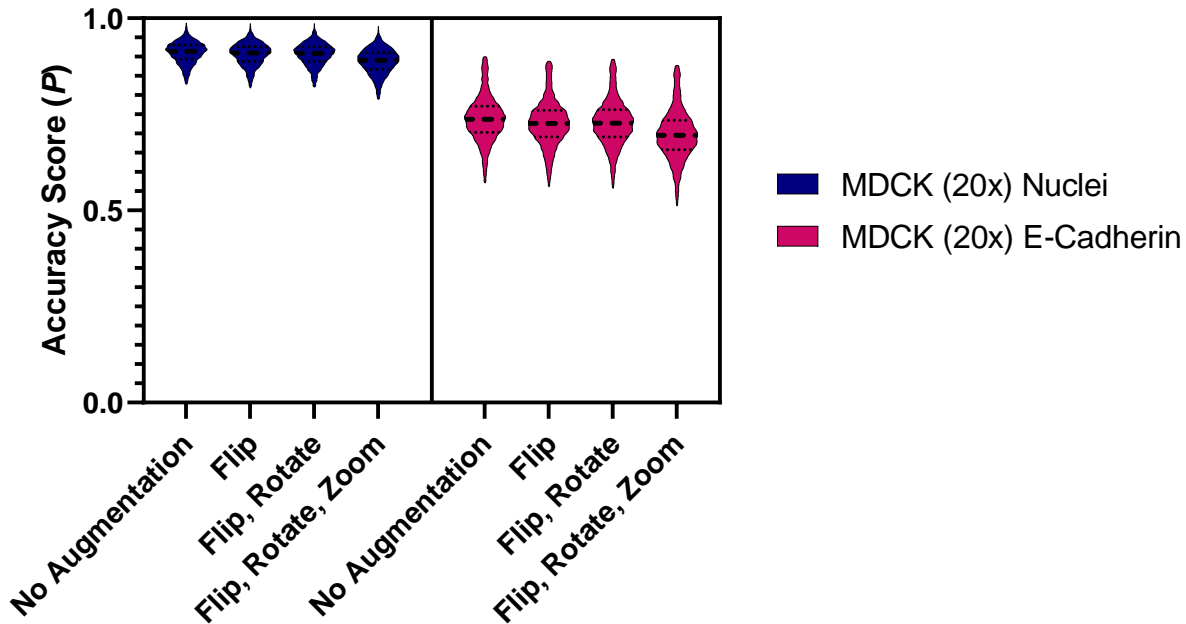

Supplement: S5 Fig — Training conditions were otherwise unchanged, and results are reported on the complete unmodified test set for each experimental condition. N = 10,000 sub-image patched for each test condition. (PDF) [file pcbi.1008443.s005.pdf]

# Training Loss Function and Accuracy

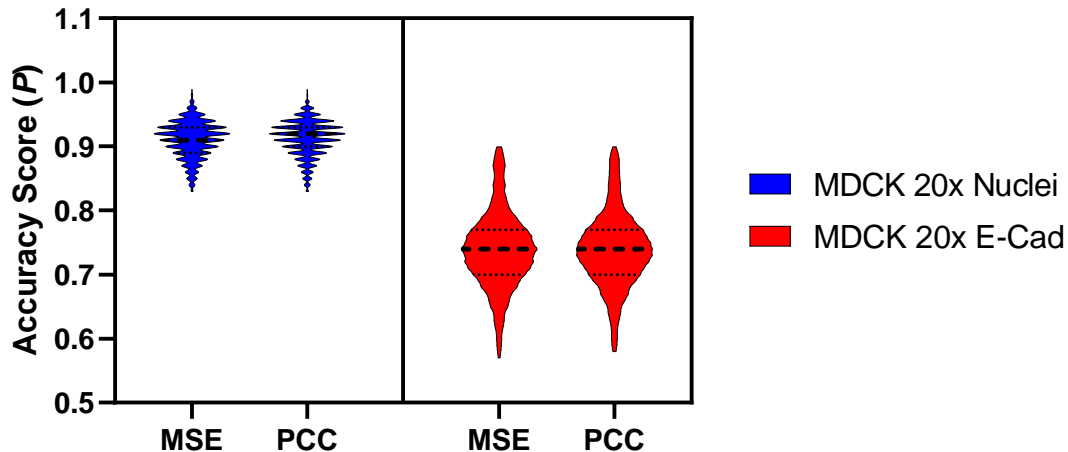

Supplement: S6 Fig — The neural network architecture and training conditions are the same, with the exception of the choice of loss function. Accuracy scores, as reported in terms of the modified P (see Methods) were comparable. N = 10,000 sub-image patches for each test condition. (PDF) [file pcbi.1008443.s006.pdf]

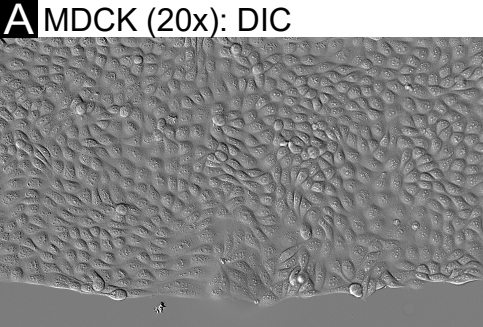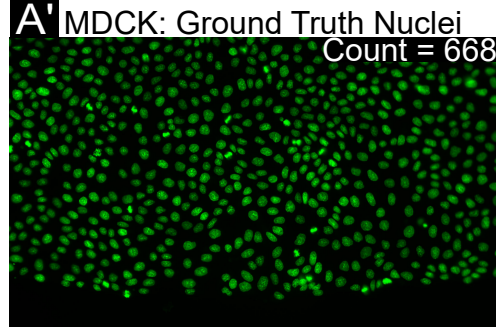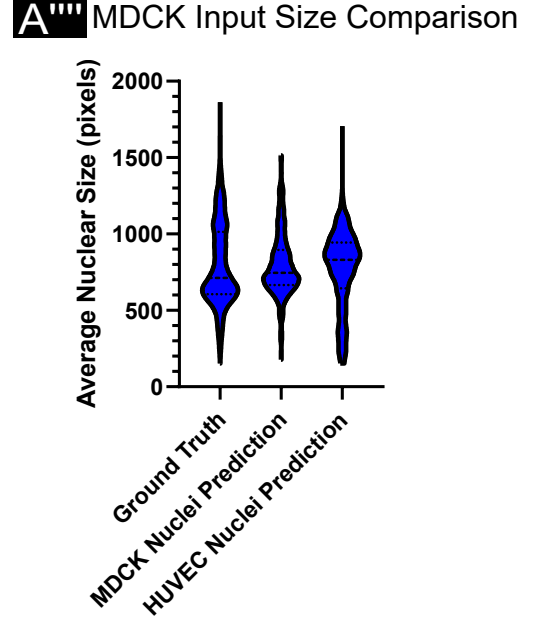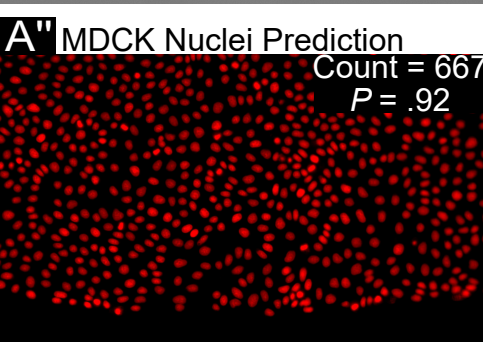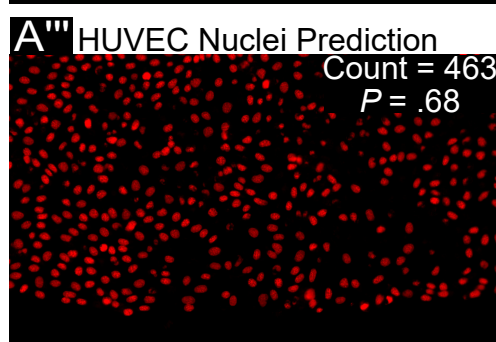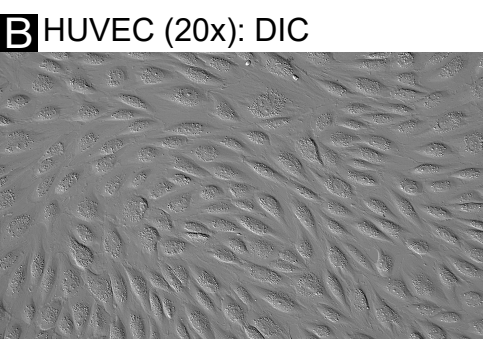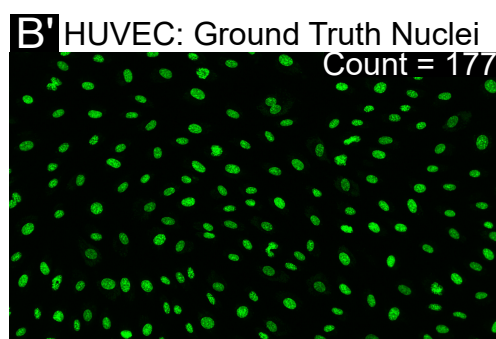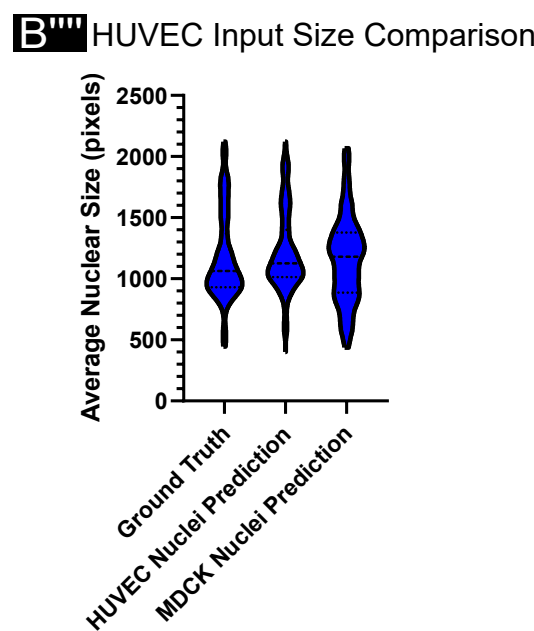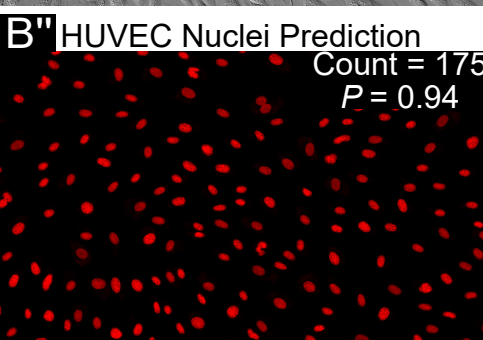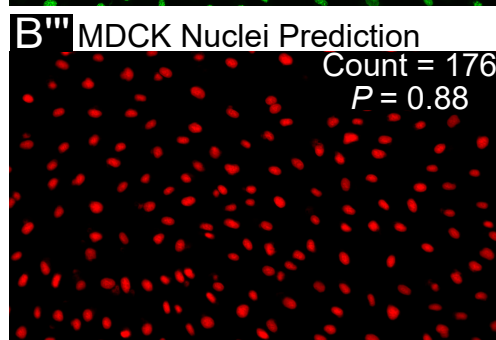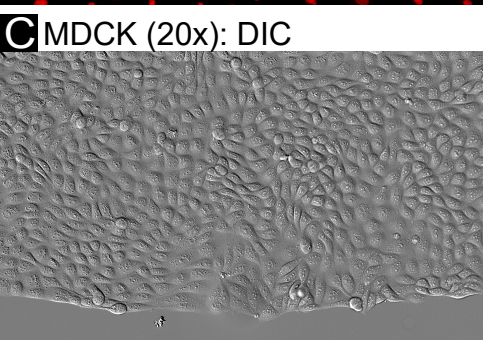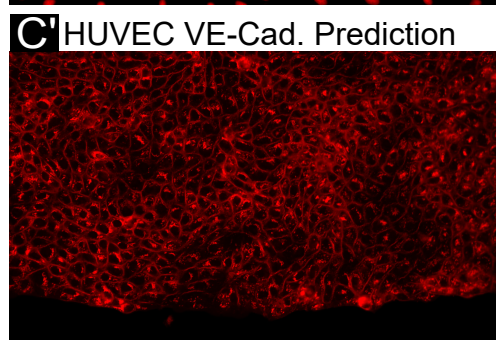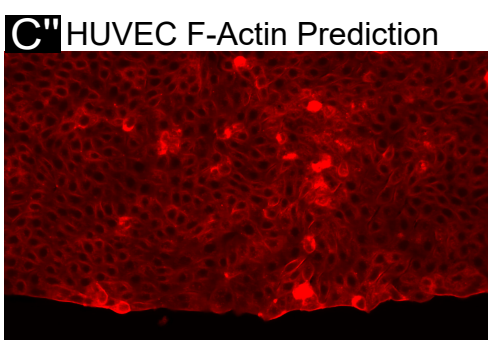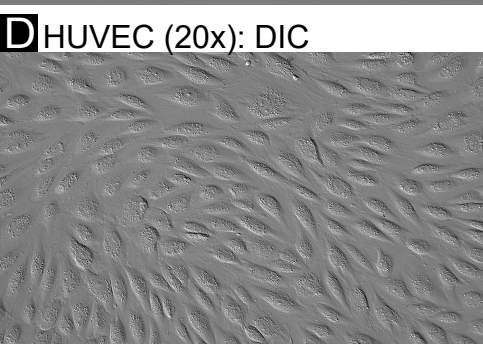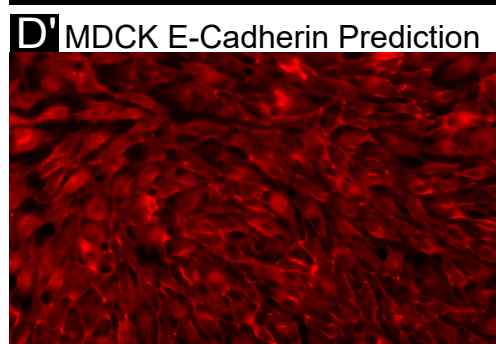

Supplement: S8 Fig — (A, B) Representative images are shown for either the MDCK cell line or the HUVEC cell line, imaged at 20x magnification. (A’, B’) For each input image, the ground truth nuclei are shown, (A”, A”‘, B”, B”‘) along with predictions from U-Net models fully trained with either the MDCK or HUVEC nuclei data, (A”“, B”“) with corresponding violin plots shown to display average nuclear size, respectively. (C, D) Additionally, the process is repeated for biomarkers lacking a training set across cell types, such as (C’, C”) VE-cadherin and F-actin predictions produced from MDCK input, or (D’) E-cadherin prediction produced from HUVEC input. (PDF) [file pcbi.1008443.s008.pdf]

# Splitting Time

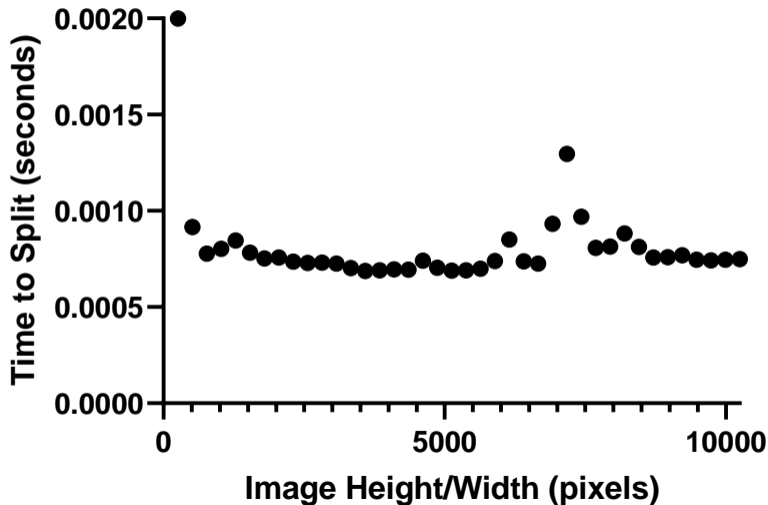

Supplement: S9 Fig — The time in seconds it takes to chop a raw image of equivalent height/width (in pixels) into 256x256 pixel2 sub-images. (PDF) [file pcbi.1008443.s009.pdf]

## Training and Validation Loss: Keratinocyte 10x Nuclei

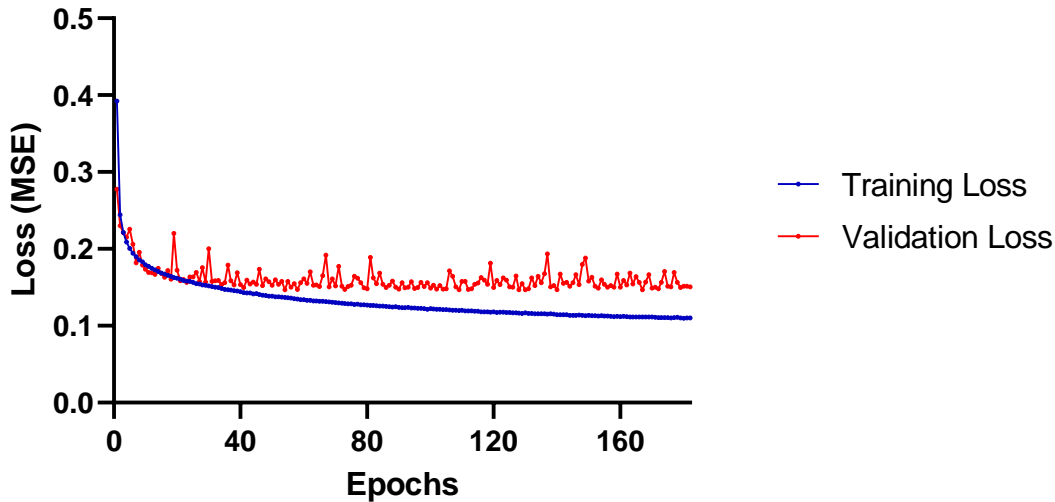

Supplement: S10 Fig — Early stopping was enabled, so that if the validation loss did not decrease within a set number of epochs, the training process terminated. (PDF) [file pcbi.1008443.s010.pdf]
