# Supplementary material for: Practical fluorescence reconstruction microscopy for large samples and low-magnification imaging
Source: PLoS Comput Biol. 2020 Dec 23;16(12):e1008443. doi: 10.1371/journal.pcbi.1008443 (PMC7802935; doi:10.1371/journal.pcbi.1008443)
Supplement: S2 Fig — (A, B) The PCC score for the representative “Ground Truth” and “Prediction” images shown in (C, D), respectively, assessed on those ground truth pixels which exceed an intensity threshold value, as the intensity threshold varies. (A) details the segmentation PCC vs. intensity threshold for an MDCK nuclei example, while (B) details this for an MDCK E-cadherin example, both at 20x magnification. The E-cadherin results do not vary smoothly with intensity threshold. (C, D) Matching ground truth and prediction images for MDCK 20x nuclei and E-cadherin examples, respectively. Segmentation masks are shown corresponding to three different intensity threshold levels. According to the choice of intensity threshold, pixels representing sub-cellular features may be excluded or undesirable background pixels may be included. (E) Three accuracy scores (PCC, P, and segmentation PCC) were compared across three datasets (MDCK nuclei at 5x and 20x magnification, and MDCK E-cadherin at 20x magnification). Outliers were not removed. (PDF) [file pcbi.1008443.s002.pdf]

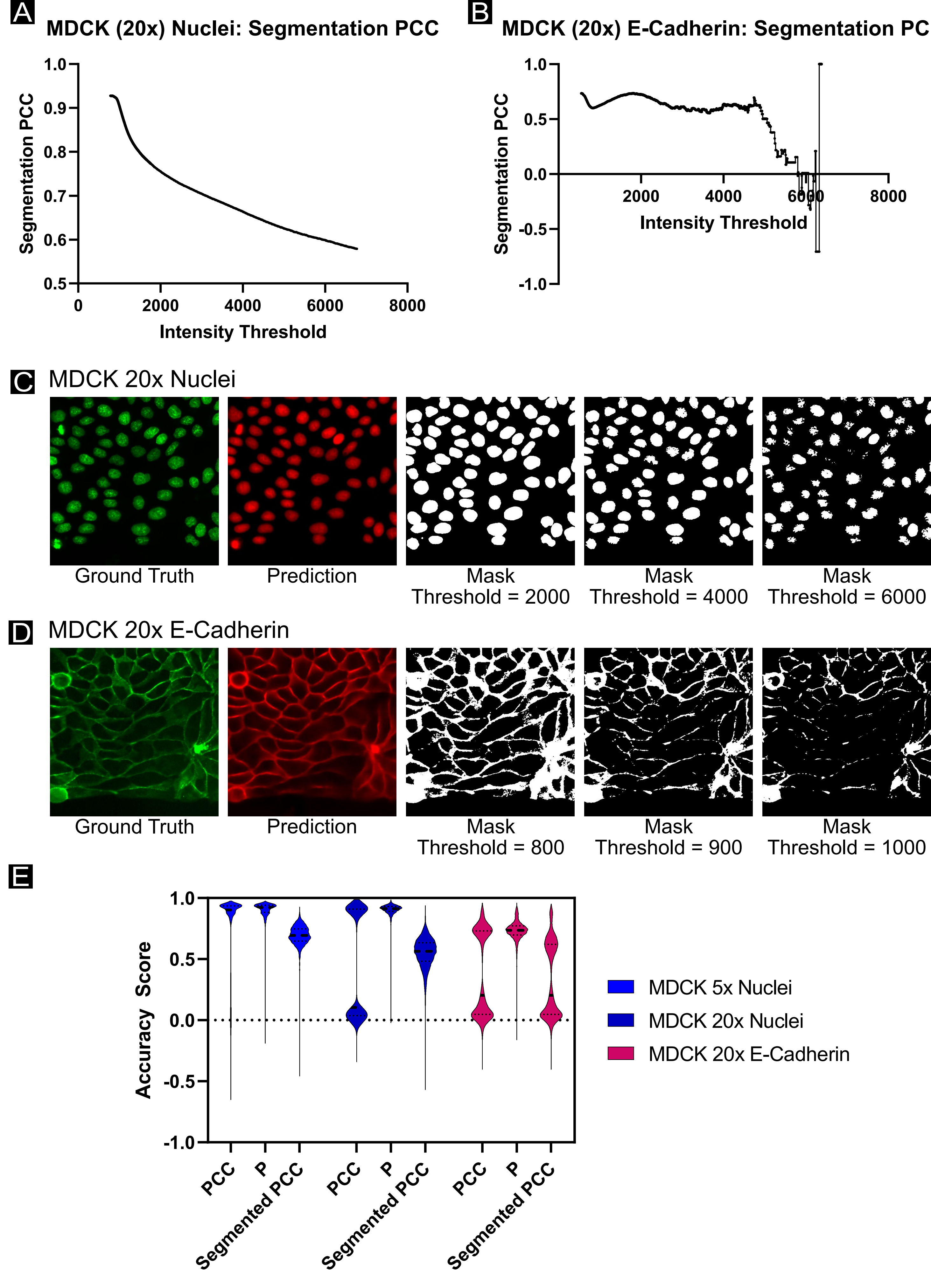
A
MDCK (20x) Nuclei: Segmentation PCC
Segmentation PCC
1.0
0.9
0.8
0.7
0.6
0.5
0
2000
4000
6000
8000
Intensity Threshold
B
MDCK (20x) E-Cadherin: Segmentation PC
Segmentation PCC
1.0
0.5
0.0
-0.5
-1.0
2000
4000
6000
8000
Intensity Threshold
C
MDCK 20x Nuclei
Ground Truth
Prediction
Mask
Threshold = 2000
Mask
Threshold = 4000
Mask
Threshold = 6000
D
MDCK 20x E-Cadherin
Ground Truth
Prediction
Mask
Threshold = 800
Mask
Threshold = 900
Mask
Threshold = 1000
E
Accuracy Score
1.0
0.5
0.0
-0.5
-1.0
PCC
P
Segmented PCC
PCC
P
Segmented PCC
PCC
P
Segmented PCC
MDCK 5x Nuclei
MDCK 20x Nuclei
MDCK 20x E-Cadherin
