# Supplementary material for: Practical fluorescence reconstruction microscopy for large samples and low-magnification imaging
Source: PLoS Comput Biol. 2020 Dec 23;16(12):e1008443. doi: 10.1371/journal.pcbi.1008443 (PMC7802935; doi:10.1371/journal.pcbi.1008443)
Supplement: S3 Fig — (Full 2D Histogram panels) All pixels in the ground truth test set are plotted against all spatially corresponding pixels in the predicted (test) set according to pixel intensity values and colored by density. A perfect prediction would correspond to a heatmap with all values on the 45-degree line. All plots display raw data without any histogram normalization. Axes are representative of camera bit depth (14- or 16-bit), with some biomarkers only filling a portion of the dynamic range. (Feature Regions panels) Represents a zoomed-in view of the data plotted in the left panel, with the ground-truth axis adjusted to show intensity values above the threshold cutoff value utilized when computing P (see Methods). This demonstrates the prediction correspondence in the positive-feature region, with low-intensity values (including background noise) excluded- which typically comprise the majority of the test set. (PDF) [file pcbi.1008443.s003.pdf]

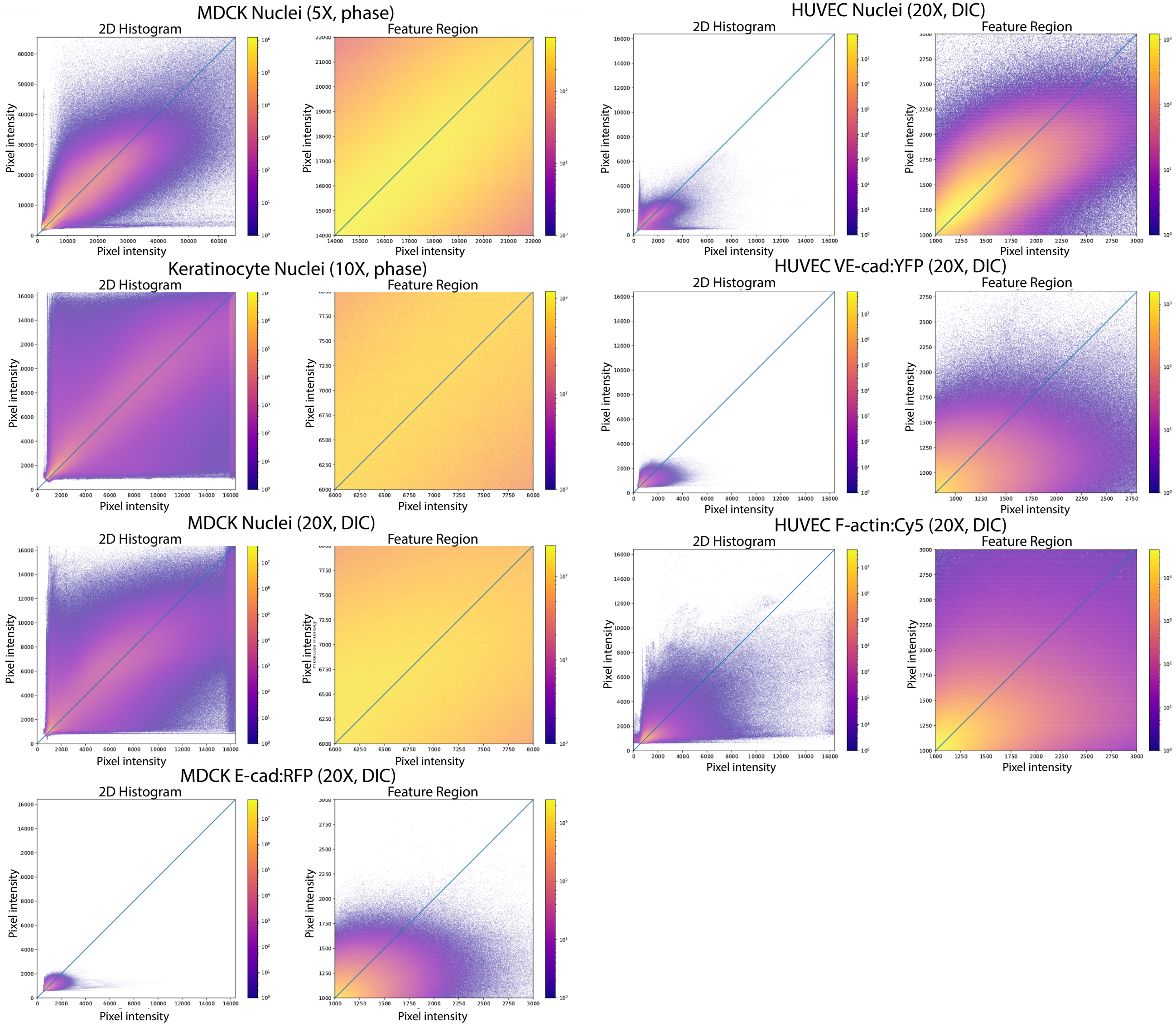
MDCK Nuclei (5X, phase)
2D Histogram
Feature Region
HUVEC Nuclei (20X, DIC)
2D Histogram
Feature Region
Keratinocyte Nuclei (10X, phase)
2D Histogram
Feature Region
HUVEC VE-cad:YFP (20X, DIC)
2D Histogram
Feature Region
MDCK Nuclei (20X, DIC)
2D Histogram
Feature Region
HUVEC F-actin:Cy5 (20X, DIC)
2D Histogram
Feature Region
MDCK E-cad:RFP (20X, DIC)
2D Histogram
Feature Region
Pixel intensity
Pixel intensity
