# Supplementary material for: Practical fluorescence reconstruction microscopy for large samples and low-magnification imaging
Source: PLoS Comput Biol. 2020 Dec 23;16(12):e1008443. doi: 10.1371/journal.pcbi.1008443 (PMC7802935; doi:10.1371/journal.pcbi.1008443)
Supplement: S7 Fig — A network was trained using data collected at 5x on a Zeiss microscope with a 6.5 μm/pixel camera, and used to process new images collected on a Nikon microscope at 4X with a 7.3 μm/pixel camera. We pre-processed data from the new system (Nikon) by scaling it to match the μm/pixel resolution of data from the original system (Zeiss) using the ‘Inter-System Correction Factor’ to rescale images in ImageJ. Representative ground truth nuclei from MDCK WT cells images on the Nikon system are shown next to the corresponding cross-platform predictions, resulting from both scaled and unscaled input images. Images were contrast adjusted for reproduction by normalizing the histograms and shifting the lower bound of the histogram up by 1/4 of the dynamic range. The scale bar represents 50 μm. (PDF) [file pcbi.1008443.s007.pdf]

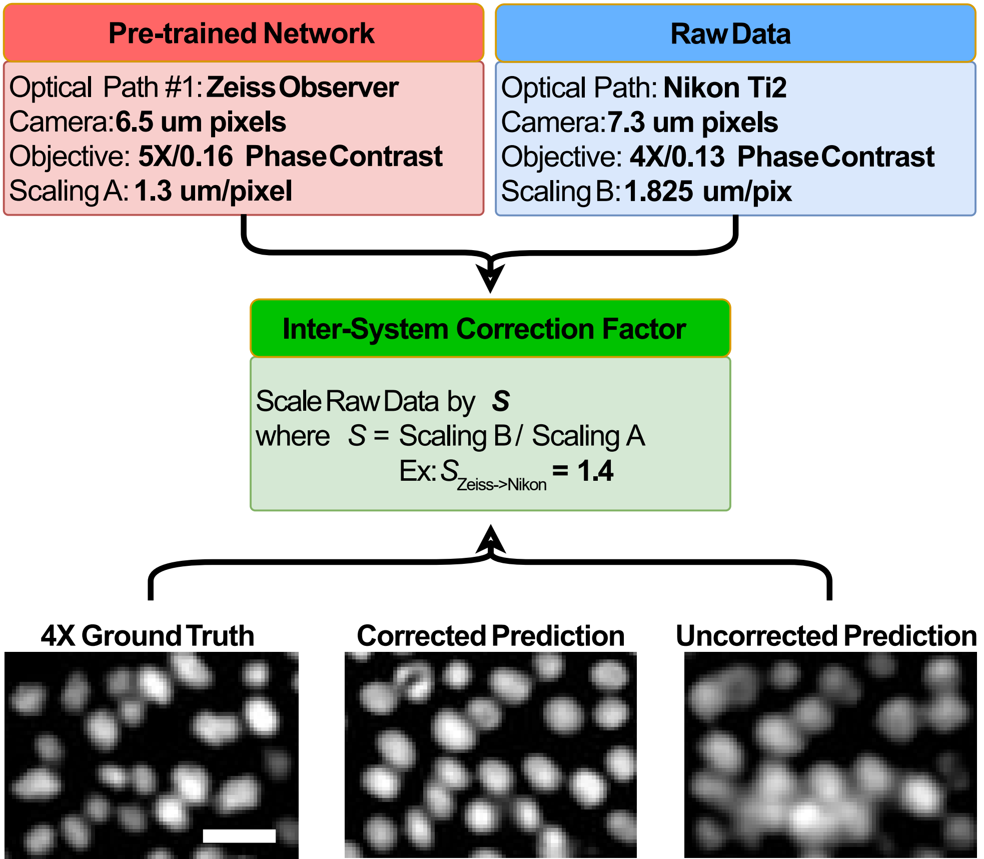
Pre-trained Network
Optical Path #1: Zeiss Observer
Camera: 6.5 um pixels
Objective: 5X/0.16 Phase Contrast
Scaling A: 1.3 um/pixel
Raw Data
Optical Path: Nikon Ti2
Camera: 7.3 um pixels
Objective: 4X/0.13 Phase Contrast
Scaling B: 1.825 um/pix
Inter-System Correction Factor
Scale Raw Data by S
where S = Scaling B / Scaling A
Ex: S Zeiss->Nikon = 1.4
4X Ground Truth
Corrected Prediction
Uncorrected Prediction
