# Supplementary material for: Practical fluorescence reconstruction microscopy for large samples and low-magnification imaging
Source: PLoS Comput Biol. 2020 Dec 23;16(12):e1008443. doi: 10.1371/journal.pcbi.1008443 (PMC7802935; doi:10.1371/journal.pcbi.1008443)
Supplement: S1 Table — (DOCX) [file pcbi.1008443.s011.docx]

| **Magnification** | **Cell type** | **Feature** | **Training Set Size (sub-images)** | **Test Set Size** | **Modified *P:* Reduced Test Set Size** | **PCC Mean** | **PCC St.Dev** | **Modifed *P* Mean** | **Modified *P* St.Dev** |
| --- | --- | --- | --- | --- | --- | --- | --- | --- | --- |
| 5x | MDCK | Nuclei | 22835 | 5709 | 4443 | 0.72529 | 0.35884 | 0.90322 | 0.07801 |
| 10x | Keratinocyte | Nuclei | 26214 | 6554 | 4156 | 0.57717 | 0.43833 | 0.9014 | 0.093155 |
| 20x | MDCK | Nuclei | 40000 | 10000 | 4556 | 0.43844 | 0.43333 | 0.90327 | 0.05724 |
| 20X | MDCK | E-cadherin | 40000 | 10000 | 4539 | 0.37466 | 0.34172 | 0.73053 | 0.091688 |
| 20X | HUVEC | Nuclei | 30720 | 7680 | 5533 | 0.68936 | 0.40728 | 0.93776 | 0.060886 |
| 20X | HUVEC | VE-cadherin | 30720 | 7680 | 5666 | 0.60824 | 0.30398 | 0.77737 | 0.074176 |
| 20X | HUVEC | F-actin | 30720 | 7680 | 5820 | 0.51247 | 0.30438 | 0.66808 | 0.14057 |
