# Supplementary material for: Practical fluorescence reconstruction microscopy for large samples and low-magnification imaging
Source: PLoS Comput Biol. 2020 Dec 23;16(12):e1008443. doi: 10.1371/journal.pcbi.1008443 (PMC7802935; doi:10.1371/journal.pcbi.1008443)
Supplement: Attachment — Submitted filename: Review_PLoSCompBio_DLFoluorescenceReconstruction.pdf [file pcbi.1008443.s018.pdf]

Review report of the manuscript “Practical Fluorescence Reconstruction for Large Samples and Low-Magnification Imaging”

Recent work have demonstrated that generative models can be trained to map label-free images to intracellular organelle localization. While relaying on the exact localization and protein expression of an organelle still is very limited and far-fetched, these approaches hold great potential for post-processing analysis tasks, such as cell segmentation and cell tracking. The potential of label free approaches for post-processing tasks stems from simplifying experimental setup, freeing fluorescent channels, reducing phototixicity (beautifully demonstrated in Fig. 2L-M) and enabling of extracting more information from old experiments. While the quantitative assessment of label-free-to-fluorescent mapping was previously based on correlation-based metrics, LaChance and Cohen propose in this manuscript that application-specific evaluation metrics should be preferred. Thinking about what is the right way to systematically assess these new techniques in a meaningful manner is an important technical issue.

The authors performed a set of imaging experiments under different objectives, cell systems and fluorescent markers, and demonstrate that they can convincingly learn a mapping from label-free to a nuclear, actin and cell-cell junctions markers. The manuscript provide solid data, is clearly written and is easy to follow. The fact that the code and the data used in this study are both organized and publically available is also an important contribution.

The manuscript is solid and deals with an important technical issue and thus I recommend publication. However, I feel that the main message of proposing “practical” evaluation measures is not fully convincing with the current analysis - some of the data presented is anecdotal and a direct mapping between the accuracy score and “practical” measures is missing. I highly recommend the authors to convey this key point with some more systematic analysis which I think will significantly improve the manuscript.

- Systematic extraction of “practical” measures:
    - In Figure 2 (nuclear markers) that authors can include a measure for the number of nuclei identified by the segmentation of the ground truth vs. the generated fluorescent channel. This is a standard readout in many applications.
    - In Figure 3 (cell-cell junctions) the data is anecdotal (line scans) and not very convincing. I would propose segmenting the fluorescent channel, measuring a “practical” readout such as cell count/size, and comparing the ground truth versus the generated fluorescent channel.
    - Matched analysis. The analysis of “practical” measures compare downstream analysis of computationally generated versus ground truth fluorescent markers. Instead of using boxplots it would be more informative to show the matched agreement between the ground truths and predicted readouts. This can visualize it as a 2D distribution/heatmap, where data close to the diagonal y = x imply high agreement.

- More systematically measure the relations between the accuracy score and the practical down-stream analysis readouts.
    - In Fig. 5B the authors demonstrate systematically the effect of training size on their accuracy measure. I would highly recommend correlating the accuracy score to “practical” down-stream analyses readouts such as cell/nuclei number and/or size. This will provide quantitative information regarding the relation between the correlation and the “practical” measures.
    - Another alternative/complementary idea for mapping the accuracy score and a “practical” measure would be to train a network on a large dataset, take several models throughout different stages of the training convergence, and then correlate the accuracy score to the corresponding “practical” measure.
- Generalization/transfer. The ability of using a trained model on a new dataset that was never seen during training is very important for the wide use of pre-trained models.
    - Several recent papers have demonstrated feasibility. For example, Christiansen et al. evaluated label-free-to-fluorescent on new cell systems, several image restoration / super resolution papers showed that their models can perform reasonably well on unseen data, and even my own recent preprint trained a model using PDX melanoma cell systems and performed a final validation with blind classification of cell lines systems. This generalization or transfer is briefly discussed in the Discussion (line #343) and in Figure S5, and I think this discussion could be extended by mentioning previous work.
    - In order to make the argument of using this approach to re-analyze legacy data, while pushing again the idea of using “practical” measures, I propose that the authors apply the HUVEC model on the MDCK data and vice versa (Fig. S5) and compare the accuracy in the resulting cell count after segmentation, using the ground truth and the matching generated fluorescent channel. The same idea could work for visualizing MDCK /HUVEC Ecad/VE-cad on the other cell model without ground truth, just for visual assessment.

<u>Additional comments and suggestions</u>:

- The idea that the goal was to compare the performance of “down-stream” analysis of the true fluorescent channel to the generated alternative can be more explicitly and clearly explained in the beginning of the results.
- Did the authors consider how the accuracy/”practical” measure deviates between imaging days? Label-free imaging can be very sensitive to external properties, such as illumination and microscopy setting that have no effect on the actual cell function. A nice validation would be training a model on several days and then testing it on a “blind” day that the model had no access to during training.

- The accuracy score (P) was determined based on an arbitrary threshold (Methods). Can the authors come up with an objective way to define this parameter?
- Other minor issues:
    - "Accuracy score" the legend of Fig. 1. I suggest to provide a brief explicit description in the legend. I know it appears later in the text, but as a fast reader going through the figures first, the lack of definition confused me.
    - Line #52 "Z-stacks of images to compute 2D reconstruction". I recall the reconstructions were 3D. Please verify.
    - Line #52 "more recently". In fact, the Ounkoml paper came out before the Christiansen paper, if I remember correctly.
    - Line #59: reference #20 is not relevant in this context.
    - Line #210 "..can be found in our supplemental data repository for comparison", please refer to the specific SI data directly.
    - Line #270: it is Fig. 5B and not 5A.
    - Line #333 reference Fig. 6, while there are only 5 figures.

Assaf Zaritsky, Ben-Gurion University of the Negev
