# Supplementary material for: Practical fluorescence reconstruction microscopy for large samples and low-magnification imaging
Source: PLoS Comput Biol. 2020 Dec 23;16(12):e1008443. doi: 10.1371/journal.pcbi.1008443 (PMC7802935; doi:10.1371/journal.pcbi.1008443)
Supplement: Attachment — Submitted filename: FRM_LaChance_Cohen_Reviewer_Responses_r11.docx [file pcbi.1008443.s019.docx]

**Revisions to *LaChance and Cohen, Fluorescence Reconstruction Microscopy*

Submitted to PLoS Computational Biology; # PCOMPBIOL-D-20-01123**

We appreciate the detailed and thorough feedback from both reviewers and have endeavored to address all review comments, both major and minor.

All reviewer comments are addressed here, with reviewer text *italicized* and our responses highlighted in **blue.** For clarity, we have numbered specific reviewer requests--1.3 = Reviewer 1, Request #3. Updates to the manuscript itself are similarly highlighted in **blue** in the revision document**.**

**Our ‘marked up’ revised manuscript was uploaded as a ‘Related File’ and is in PDF format with figures included to make it easier to review. The line numbers listed below correspond to this document and not to the ‘clean’ copy.
See file: FRM_LaChanceCohen_ChangesHighlighted.pdf.**

**Reviewer #1:**

**Major Concerns.**

*One of the main focuses of the paper is how to properly benchmark FRM methods. This is important, and the author’s highlight significant shortcoming in the PCC metric. This metric suffers from a classic issue with data centered benchmarking - it is overwhelmed by easy examples. In this case, the authors argue (rightly so) that the “easy” example is the background. A model that can distinguish background from foreground will falsely report a high PCC because the existence background and foreground will effectively induce a correlation (similar to Simpson’s paradox). Benchmarking datasets that have high numbers of background pixels will be prone to this bias. The authors offer an alternative metric - the corrected accuracy score - in which the PCC is computed only on images that have positive examples of the structures in question.*

**1.1:** *This approach has two issues. First, this appears to be a half measure. I believe a better correction would be to segment foreground from background pixels and then compute the PCC only on the foreground pixels. Choosing images with only positive examples doesn’t resolve the issue, because those images can still contain background pixels. Computing the PCC on the foreground (e.g. cellular) pixels would resolve this issue, as most uses of FRM seek to resolve subcellular structures.* ***Annotation of foreground/background pixels - either manual or computational - should be performed, and the data reanalyzed with these annotations take into account. If this approach is successful, the authors should explicitly define a new metric in their resubmission, to show the connection to and departure from PCC.***

**Response to Request #1:**

We agree that the PCC is a problematic metric and it is interesting to further augment our corrected PCC (which we call *P*) as suggested based on segmented pixels. To address the reviewer concern, we completely re-analyzed three of our key datasets in response to the suggestion, as shown in the new Fig. S2. Here, we will re-explain the importance of our original current *P* metric, and then compare this to the approach suggested by the reviewer, which we call ‘Segmentation PCC”.

*P*, our original corrected PCC value, is based on the PCC values from the subset of images which were determined to contain positive examples of features, as a function of a fixed intensity threshold. Those background images which were removed from analysis by this metric result in misleading PCC scores when the entire dataset is analyzed. This is because the pseudo-random background noise which the network attempts to produce does not align with the true background signal in the ground-truth images; so the PCC scores vary widely. Thus, our modified PCC score restricts the analysis to cut out those background-only images.

Reviewer #1 suggests that we go further in correcting the standard PCC to incorporate what we here call ‘Segmentation PCC’. This metric emphasizes subcellular features and provides new information about the success of the model to reconstruct features. Specifically, while our current *P* was designed to capture information about how well the foreground is distinguished from the background, the Segmentation PCC would instead provide information about how well sub-features are made distinct within larger structures. This is an interesting use case, and we now provide a full analysis of the performance of the Segmentation PCC on three of our key datasets for comparison--see **Fig. S2.**

However, we had a number of concerns with the performance of the Segmentation PCC as a general metric for FRM, and we were hesitant to re-analyze the entire paper using the Segmentation PCC. Specifically, we have two key concerns we observed. (1) The Segmentation PCC is straightforward to implement when an image can be unambiguously segmented (e.g. nuclei), but is difficult to apply when the information to be analyzed is spread more widely across a grayscale spectrum, (e.g. variable intensity E-cadherin junctions, projected 3D features) where segmentation masking is difficult to standardize. (2) If the user is primarily concerned with distinguishing general foreground from background rather than distinct subcellular features, our *P* may be more relevant.

As an example of the latter case (1), please consider Fig. S2 (D), displaying representative E-cadherin images, and segmentation masks at different threshold levels. Intensity thresholding is inherently based on subjective user decisions, and the graded intensity E-cadherin levels in junctions makes it unclear where the cutoff should be when assessing the Segmentation PCC. A generous threshold (low intensity value) may include too much of the background in certain regions, thus presenting similar problems to *P*, while a restrictive threshold (high intensity value) prevents the metric from capturing information about the performance of the model in regions where the junctions are short. In Fig. S2 (B), we provide the segmentation PCC values which correspond to the representative images below, and how they vary as a function of the segmentation threshold. Fig. S2 (A, C) provide the same for the nuclei dataset for the equivalent cell type and magnification level.

Nonetheless, the Segmented PCC is clearly useful for certain user applications, so we included discussion of the new figure in Lines [89-97], as follows:

“*We also considered a second modified metric, which we call “Segmentation PCC”, which reports PCC only on those pixels which exceed an intensity threshold in the ground truth image (Fig. S2, see Methods). However, since many of our key datasets either (1) are used in applications which require background information, such as with nuclei segmentation/tracking, or (2) represent structures on the grayscale spectrum where the background/foreground distinction is highly subjective, such as with MDCK E-cadherin, we found the P metric to be more suited for our analyses. The Segmentation PCC may benefit future researchers in assessing foreground accuracy in alternative FRM applications*.”

We also updated the Methods section, with modifications in Lines [554-560]:

“*...unless otherwise stated. The Segmentation PCC score is determined by using the same intensity threshold used in the P analysis, and determining which pixels in each ground truth image exceed that intensity value in order to produce a mask image. Then the standard PCC is reported against the pixels in the ground truth image which correspond to that mask and the pixels in the prediction image which correspond to the mask, and this process is repeated across the entire test set.*”

**1.2:** *The second issue is that FRM does require annotation. The need for annotated training data has been a significant barrier to the adoption of deep learning methods in the life sciences, and the reluctance to embrace this challenge has actually shaped the space of methods developers. While FRM is a great method, the reason why it is popular is because it has a low annotation burden. It only requires the collection of image pairs (label free and fluorescence), and does not need dense pixel level annotations for model training. As this paper reveals, however, annotation of some form is necessary for benchmarking. Without proper, unbiased benchmarks it is impossible to know what is an advance and what is not.* ***Commentary on this point would make the paper considerably stronger****, as it would better clarify the weakness of PCC as a metric and why better metrics have not been developed.*

**Response:**

We agree that this is an important issue to raise, so we have included additional commentary in the discussion section to highlight issues facing the field with regard to proper benchmarking. We added a new section beginning at line 373 as follows:

*“Our work also highlights how FRM and similar machine learning techniques need performance benchmarks and metrics that are both standardized and, critically, context-aware. For instance, although FRM has a relatively low annotation burden (as the fluorescent channel essentially auto-annotates the transmitted light channel) for training purposes, there still remains the problem of how the user knows how a given FRM prediction performs against benchmarks of practical value to that user. Critically, we demonstrated here that common, but abstract, benchmarks often fail to clearly reflect the practical value of that FRM reconstruction for the average biologist. In other words, what is ‘mathematically good’ (e.g. high P, etc.) does not adequately reflect what is ‘practically useful’. Hence, while FRM is clearly powerful, it is like any other tool and requires that the user deploy and assess it intelligently. Establishing clearer benchmarks, challenges, and more approachable performance metrics would be of great value towards transitioning FRM and similar techniques into standard practice.”*

**1.3:** *The usability of the software package is less than what I would have expected for a software paper. A Dockerfile should be included, and a pre-built docker image should be posted on Dockerhub. The dependencies are given but are not versioned; this makes setting up a working installation significantly harder. Moreover, the software pipeline as described is (in my view) unwieldy. Key steps for processing data are separated out into different languages (image cropping in fiji, model training/processing and image stitching in python). I feel like this should be done all in one language - a TensorFlow add-on to ImageJ exists, while image cropping is straightforward to handle in python. If the authors choose to go the python route, object oriented programming would be an effective way to organize the different operations that have to be applied to an image (e.g. image resizing, normalization, cropping, model processing, stitching). A GUI would make a Python package more useable (see e.g. NuSet from Jan Liphardt on biorxiv) but wouldn’t be a hard requirement for resubmission. Barring that, a single pre-executed Jupyter notebook accessible on the repository’s README demonstrating analysis with a pre-trained model would be a considerable improvement.*

**Response:**

We agree that usability needed to be improved, so we have implemented each of the Reviewer’s suggestions as follows:

1. Per the suggestion to keep tools within a single language, we have created versions of the code corresponding to every part of the pipeline in Python. (The old ImageJ/Jupyter notebook files are also provided as an alternative, in case the user is more comfortable performing image pre-processing using ImageJ, or prefers the flexibility of the Jupyter notebook for viewing output.)
2. We created a single pre-executed Jupyter notebook which shows the user how to use a model and pre-trained weights file to process a single small image (with a sample image provided and output displayed in the notebook).
   <https://github.com/CohenLabPrinceton/Fluorescence-Reconstruction/tree/master/How_To_Use_The_Model>
3. We produced a more streamlined option for users who simply want to train a new model on their own data without looking at the code. A bash script is provided to install all required Python libraries locally, and the user just needs to edit a few simple parameters (e.g. paths to raw image data) and run a single Python script, called “main.py”, here:
   <https://github.com/CohenLabPrinceton/Fluorescence-Reconstruction/tree/master/Lab_FRM>
   We additionally provide a second script, called “process_new_images.py”, if users want to then apply the newly trained model to new images.
4. A sample Dockerfile was produced which demonstrates how to install all the required Python libraries required to run our code in an Ubuntu image. Users may either install the required Python libraries on their own (with library versions provided in the User Manual), or use the commands given in the Dockerfile to perform the update/install. Savvy users may utilize the sample Dockerfile to create their own image for running the code; we provide a very simple Python example as well as build/run commands for executing the test script. We do note that it is important for users to install the necessary drivers for their own hardware (e.g. CUDA), which is why we are uncomfortable about a one-size fits all docker file. That said, our objective was to keep the Python code self-contained within each part of the pipeline and readable to novice programmers. The Docker tools are given here: <https://github.com/CohenLabPrinceton/Fluorescence-Reconstruction/tree/master/Dockerfile>
5. We also field tested our FRM approach with several researchers who had no prior experience in Python or machine learning. They have been able to successfully use it for their own data sets now.

**Minor Issues from Reviewer #1:**

**1.4:** *The analysis of model performance vs dataset size is interesting, but it would be more useful if this was couched as # of cells vs # of images. This should be straightforward to compute given that brightfield images can be converted into nuclear images. The comment that this relationship is non-linear is somewhat unnecessary, as non-linearity is implied by the PCC and the corrected accuracy score being bounded above by 1. More important is the monotonicity of the relationship - while there are diminishing returns, better data leads to better models.* ***This should be highlighted in the paper.***

**Response:**

We adjusted the caption in Fig. 5B to reflect the # of cells/image. The caption now includes the text:

“*The average number of cells per sub-image were: MDCK 5x: 107 cells per sub-image; Keratinocyte 10x: 26 cells per sub-image; MDCK 20x: 11 cells per sub-image; HUVEC 20x: 3 cells per sub-image.*”

We also updated the main text to more clearly reflect that more/better data leads to better models and highlight the monotonic relationship shown in Fig. 5 between data/training and performance.

Lines 289-295 now read: *“The relationship between P and training set size is shown in Figs. 5A,B, where we show both how a single camera images is first chopped into 16 (typically) sub-images for analysis (Fig. 5A), and how P and training set size vary relative to either camera images or sub-images (Fig. 5B). These data demonstrate that the rate of change in quantitative quality (P) vs. training set size is monotonic across different biomarkers, with more data yielding higher accuracy scores with diminishing returns.”*

**1.5:** *Training for the model performance vs dataset size analysis was performed without data augmentation. While this is understandable for reproducibility, it makes the analysis considerably less informative, as data augmentation can increase the diversity of a given dataset. This analysis should be redone with proper augmentation (rotation, reflection, and zoom) and presented. The non-augmented analysis could be removed or placed in the supplement, as the comparison would allow readers to see the value of augmentation.*

**Response:**

We agree that showing the effect of data augmentation is valuable, and demonstrate the results in Fig. S5 on two key datasets. Performing reflection, rotation, and zoom to augment the dataset were determined to offer little to no benefit to a model trained on a standard U-Net architecture in this context, so we did not re-do all analyses in the paper--perhaps because our dataset size was already sufficiently large and varied. However, as augmentation is generally useful we have added a note in the discussion as shown below:

Data augmentation was addressed main text in Lines 342-345:

*“While no substantial benefit was observed as the result of data augmentation (flip, rotation, zoom) on standard U-Nets trained with large and varied datasets (see Fig. S5), users should test data augmentation techniques on their own data to assess accuracy gains prior to widespread application.”*

With details added to *Methods* in Lines 534-539:

“*Likewise, for several experimental conditions, data augmentation was applied in the form of flipping (both horizontally and vertically), rotation (in increments of 45 degrees), and zoom (up to 20% scale). For flips and zoom, built-in Tensorflow functions were utilized. However, the raw data was re-processed to apply random rotations in order to avoid boundary problems using ImageJ/FIJI, resulting in a modified training set which was five times the size of the original dataset.*”

**1.6:** *The importance of spatial scale for model performance has been described previously in the context of segmentation (see Van Valen et al PLOS CB 2016 and the recent NuclAIyzer paper from Peter Horvath’s group Cell Systems 2020). One of these works should be cited and this insight should be placed in context.*

**Response:**

We agree that this is a valuable discussion, and have added a note about receptive field size optimization, based on Van Valen et al (PLOS CB 2016), in the main text in Line 67:

“*Van Valen et al (PLOS CB 2016) examine the trade-off between conv-net receptive field size on feature segmentation accuracy versus training time (and therefore over-fitting of the model): optimization of the receptive field size may lead to further performance gains.*”

**1.7:** *Pre-trained models should be made available via TensorflowHub.*

**Response:**

We have submitted the pre-trained model for each experimental condition to Tensorflow Hub with documentation in accordance with the Tensorflow Hub protocol. However, we were not aware that Tensorflow Hub is a relatively new repository with an internal approval process and our submission is relatively low in a back-logged queue. We hope this does not affect review of our manuscript here.

**1.8:** *Benchmarking of inference speed (images/second for 1-megapixel images) for the model should be reported. This should also include the time required to crop and stitch images*

**Response:**

We include the image cropping time data as a function of image size in Fig. S8, and training times as a function of number of epochs for three key datasets in Fig. 6. The processing and stitching protocol times were discussed in the Method sections of the main text as follows.

Methods section, Lines 505-507, now read:

*“The splitting times are reported as a function of image size (see Fig. S8); this plot was generated by cropping the same image into smaller TIFF images, and applying the same splitting procedure to chop the image into 256x256 pixel^2^ sub-images. Input images were then...”*

And in Lines 527-528:

*“...and timing data as a function of epochs is given for key datasets (see Fig. 6).*”

And finally, in Lines 590-593:

*“On a standard desktop with an NVIDIA GeForce GTX 1070 Ti GPU, the time to read in, process, and save out a single 256x256 pixel^2^ image was 0.49 seconds, and the time to read in, split, process, stitch, and save out a 1024x1024 pixel^2^ image was 10.3 seconds.*”

**1.9:** *Source code should contain a Dockerfile.*

**Response:**

We have provided installation tools and address this point more fully in the response to reviewer comment 1.3.

**1.10**: *A description of the deep learning software stack (e.g. Tensorflow/Keras vs Pytorch) should be included.*

**Response:**

We clarified our decision to use Keras/Tensorflow in the Methods section. Line 512-515 now reads:

*“The U-Nets shown in Figure 1 were implemented and trained in Python using the Keras/Tensorflow packages, since we find these to be more researcher-friendly for high-level deep learning implementations than alternative open-source deep learning libraries (e.g. PyTorch). Training was performed using the Tensorflow ADADELTA optimizer.”*

**1.11**: *Figure 2 L-M was hard to see - it would be probably best to move this to a supplement so they can be seen at full size*

**Response:**

To view the figure at full size, readers may refer to Movie S1. The figure caption text for Figure 2 (now 2M,N) was updated to reference this movie more clearly, and now reads:

*“Higher resolution movies showing the migration dynamics can be seen in Movie S1.”*

**1.12**: *While the authors claim model architectures with more capacity (e.g. residual U-Net) did not provide a boost in performance, it would be interesting to explore models with reduced capacity. If a mobilenet/FPN was used for feature extraction instead of the conv/max pool from U-Net, would the performance be compromised? Models that are lighter weight but retain accuracy are more valuable, as they are more scalable and can have reasonable throughput if used on CPUs.*

**Response:**

This is an important question for future work in the community, but repeating all of our analyses with a different architecture felt outside the scope of this paper. We do, however, hope others explore this avenue as it will be of benefit.

**Reviewer #2:**

**Major Concerns.**

*Systematic extraction of “practical” measures:*

**2.1:** *In Figure 2 (nuclear markers) that authors can include a measure for the number of nuclei identified by the segmentation of the ground truth vs. the generated fluorescent channel. This is a standard readout in many applications.*

**Response:**

We have provided measures of segmented nuclei in the form of pairwise comparison plots to visually capture counts, as can be seen in Fig. 2L.

Text update (Lines 179-181): “*Finally, we compared basic cell counts using a pair-wise comparison plot (decimated), which demonstrated a mean count error over the entire datasets between ground truth and FRM predictions of ~1.4% (Fig.2L).”*

**2.2:** *In Figure 3 (cell-cell junctions) the data is anecdotal (line scans) and not very convincing. I would propose segmenting the fluorescent channel, measuring a “practical” readout such as cell count/size, and comparing the ground truth versus the generated fluorescent channel.*

**Response:**

We have addressed this request and the resulting practical measure can be found in Fig. 3I. Briefly, our analysis of 30,000 cells revealed that E-cadherin segmentation of FRM data and ground truth data resulted in very similar areal distributions with mean calculated cell areas differing by only ~1%. We have updated the text and methods sections accordingly, as described:

Text updates (223-227): *“As a demonstration of a common practical analysis performed using junctional data, we calculated the distribution of cell areas in both ground truth and FRM predictions (see Methods). Shown in Fig. 3I, we observed very similar area distributions with mean calculated cell areas over ~30,000 individual cells varying by ~1%.”*

Methods text (572-581): *“Cell areas were calculated using either fluorescence E-cadherin junction data or FRM predictions of the same using a large image containing ~30,000 individual cells. We opted to perform analysis in entirely in FIJI to better represent what the average user might use. We performed the following steps to calculate and compare cell areas. Image pairs were first histogram-normalized to 16-bits. Images were then segmented manually using standard intensity thresholding in FIJI with the same threshold(~30% cut-off). Binary skeletonization was used on both ground truth and FRM images to produce cellular outlines. Outlines were then closed using a single dilation operation. After inverting the images, a standard ‘Analyze Particles’ in FIJI was used to calculate areas inside the boundaries. These distributions were then plotted to allow comparison.”*

**2.3:** *Matched analysis. The analysis of “practical” measures compare downstream analysis of computationally generated versus ground truth fluorescent markers. Instead of using boxplots it would be more informative to show the matched agreement between the ground truths and predicted readouts. This can visualize it as a 2D distribution/heatmap, where data close to the diagonal y = x imply high agreement.*

**Response:**

We appreciate that 2D heatmaps are a standard visualization and have provided a supplemental figure showing the complete 2D heatmaps across our entire data corpus for all analyzed conditions. We followed a similar format to Ounkomol et al. We felt it was important to leave the boxplots in as well as these are the simplest metric used in the field. 2D heatmaps can now be found in Fig. S3. The plots are referenced in the main text in Lines 111-113:

*“While we necessarily show representative data here, we provide the statistical distribution for all accuracy scores, as well as complete 2D cytofluorogram heatmaps of ground truth to prediction mapping across all of our data for each biomarker (Fig. S3).”*

**2.4:** *In Fig. 5B the authors demonstrate systematically the effect of training size on their accuracy measure. I would highly recommend correlating the accuracy score to “practical” down-stream analyses readouts such as cell/nuclei number and/or size. This will provide quantitative information regarding the relation between the correlation and the “practical” measures.* ***Another alternative/complementary idea for mapping the accuracy score and a “practical” measure would be to train a network on a large dataset, take several models throughout different stages of the training convergence, and then correlate the accuracy score to the corresponding “practical” measure.***

**Response:**

We thank the reviewer for the suggestion, and opted to both: (1) add a practical readout in the form of nuclei counts for representative images in Fig. 5 to systematically demonstrate the effect of reduced training size, and (2) perform the suggested alternative approach, measuring the nuclei count (as well as our *P* accuracy score) as a function of ***reduced epochs*** in the training process. The latter results can be found in the new figure, Fig. 6.

Edit (1) is addressed in the main text in Lines 295-299:

*“To relate these data to a more practical readout, we demonstrate how nuclear count performance depends on training set size using our 5X and 20X MDCK datasets (Figs. 5C,D). These data emphasize both that FRM performance variance goes down with larger training sets, but also that relatively small numbers of training images can produce useful results.”*

Edit (2) is addressed in the main text in Lines 318-333:

*“We next assessed how the number of training epochs (and associated convergence) affected network performance. This is valuable because some researchers may have a great deal of training data but limited computational resources. Here, we present data from several key datasets in Fig. 6 where identical U-Net models were trained for a fixed number of epochs (1, 5, 10, 50, 100, or 500 epochs) and the accuracy score (P) (Fig. 6A). Additionally, we report the actual training hours (Fig. 6B) based on our specific hardware (NVIDIA Tesla P100 GPU). Again, we relate these data to practical performance using nuclei counts, this time plotting how the distribution of counts evolve as a function of training epochs and relative to the ground truth distribution (Figs. 6C,D). Overall, increasing training epochs correlates with improved performance, as highlighted visually in Figs. 6E,F where the evolution of predictions of nuclei and E-cadherin are presented relative to training epochs. In general, researchers limited by computational bottlenecks may opt to reduce training time as the network converges to ‘good enough’ results as a function of their practical measure of performance. To emphasize this, consider Fig. 6E, where the dashed circle indicates how regions of higher cell density reconstruct poorly for low training epochs; highlighting the importance of assessing practical model performance on a range of potential biological and imaging conditions.”*

**2.5**: ***Generalization/transfer****. The ability of using a trained model on a new dataset that was never seen during training is very important for the wide use of pre-trained models. Several recent papers have demonstrated feasibility. For example, Christiansen et al. evaluated label-free-to-fluorescent on new cell systems, several image restoration / super resolution papers showed that their models can perform reasonably well on unseen data, and even my own recent preprint trained a model using PDX melanoma cell systems and performed a final validation with blind classification of cell lines systems. This generalization or transfer is briefly discussed in the Discussion (line #343) and in Figure S5, and I think this discussion could be extended by mentioning previous work.*

**Response:**

We agree that this is a valuable discussion to strengthen the main text, and we have included it.  ***See joint response under 2.6***

**2.6**: *In order to make the argument of using this approach to re-analyze legacy data, while pushing again the idea of using “practical” measures, I propose that the authors apply the HUVEC model on the MDCK data and vice versa (Fig. S5) and compare the accuracy in the resulting cell count after segmentation, using the ground truth and the matching generated fluorescent channel. The same idea could work for visualizing MDCK /HUVEC Ecad/VE-cad on the other cell model without ground truth, just for visual assessment.*

**Response:**

We applied the HUVEC model to the MDCK data, and vice versa, and have displayed the resulting predictions, plus corresponding nuclei counts resulting from segmentations of the predicted images, in Fig. S8. Nuclei predictions were reasonably conserved across networks, but other markers were far less meaningful (e.g. MDCK cells naturally lack VE-cadherin structures). We also noted this in the main text as follows in Lines 417-423:

*“We also considered the application of trained networks across different cell types; comparable cross-cell-line analyses have previously been performed^34^. Fig. S8 presents such cross-network results indicating sufficient performance for nuclei predictions that researchers unfamiliar with FRM could begin by testing pre-trained networks on their own data. As with cross-platform applications, we recommend researchers train a model using data which best captures the conditions under which they expect the model to be applied- including consistent cell types.”*

**Minor Issues from Reviewer #2:**

**2.7**: *The idea that the goal was to compare the performance of “down-stream” analysis of the true fluorescent channel to the generated alternative can be more explicitly and clearly explained in the beginning of the results.*

**Response:**

We agree that this clarification is useful, and have addressed it within the text in Lines 109-113.

*“Specifically, we apply commonly used ‘down-stream’ analyses from high-content screening to both ground truth and FRM predictions to evaluate how closely analysis of a prediction matches that of the ground truth.”*

**2.8**: *Did the authors consider how the accuracy/”practical” measure deviates between imaging days? Label-free imaging can be very sensitive to external properties, such as illumination and microscopy setting that have no effect on the actual cell function. A nice validation would be training a model on several days and then testing it on a “blind” day that the model had no access to during training.*

**Response:**

We agree that this is an important point: in fact, our sample movies [e.g. Movie S1] were captured on separate days. In lines 185-186, we note that “we input legacy data from a 24 hr time-lapse experiment of the growth dynamics of large epithelia”. Moreover, the tool we have developed has already been deployed for truly blind datasets (see Heinrich et al. eLife 2020; and Zajdel and Shim et al., Cell Systems 2020), in which image data was collected with no prior knowledge of imaging conditions from the training dataset capture.

**2.9**: *The accuracy score (P) was determined based on an arbitrary threshold (Methods). Can the authors come up with an objective way to define this parameter?*

**Response:**

We appreciate this comment, as it is a point we have considered ourselves: ultimately, thresholding methods do require some decision on the part of the user. A user might choose a reasonable representative image, run Otsu’s method (or similar) to determine the threshold, but it will still be up to the user to determine if this threshold works for their data. For our purposes, we were able to rely upon the auto-threshold method in ImageJ, which by default is a variation of the IsoData algorithm, across multiple images containing positive examples of the feature. As the average cell biologist also uses the ‘auto-threshold’, we felt this to be reasonably representative.

**2.10**: *“Accuracy score” the legend of Fig. 1. I suggest to provide a brief explicit description in the legend. I know it appears later in the text, but as a fast reader going through the figures first, the lack of definition confused me.*

**Response:**

We have addressed this by updating the legend of Fig. 1. The caption of Fig. 1 (D) now includes the text:

*“Here, the accuracy score (P) represents the Pearson’s correlation coefficient between ground truth images containing positive examples of features and their matched reconstructed images. See Methods.* ”

**2.11**: *Line #52 “Z-stacks of images to compute 2D reconstruction”. I recall the reconstructions were 3D. Please verify.*

**Response:**

In the case of the paper “*In Silico Labeling: Predicting Fluorescent Labels in Unlabeled Images*” by Christiansen et. al, the authors take in 3D z-stacks of transmitted light images as input, yet output a single 2D fluorescence reconstruction image. To produce the 2D fluorescence image from the captured z-stack, the authors use z-axis maximum projection, and “*in which the target (fluorescence) images are projected along the z-axis by taking the 90th percentile intensity as a robust estimate of the maximum*.”. They note that 3D (voxel) prediction was not done in this work, but that they’d seen promising results in that space.

**2.12**: *Line #52 “more recently”. In fact, the Ounkoml paper came out before the Christiansen paper, if I remember correctly.*

**Response:**

We thank the reviewer for noticing this error and have removed temporal dependence in the text. Lines 51-53 now read:

*“While several FRM methods use computationally complex and expensive networks that rely on Z-stacks of images to capture 2D reconstruction, other approaches reconstruct 3D image stacks using a modified U-Net architecture.”*

**2.13**: *Line #59: reference #20 is not relevant in this context.*

**Response:**

We have removed this reference in this context.

**2.14**: *Line #210 “..can be found in our supplemental data repository for comparison”, please refer to the specific SI data directly.*

**Response:**

We have added the SI data reference. Lines 229-231 now read:

*“A large dataset of reconstructed junctions and nuclei can be found in our supplemental data repository (http://doi.org/10.5281/zenodo.3783678, see Code and Dataset Availability) for comparison.”*

**2.15**: *Line #270: it is Fig. 5B and not 5A.*

**Response:**

We have adjusted the reference accordingly. Lines 289-292 now read:

*“The relationship between P and training set size is shown in Figs. 5A,B, where we show both how a single camera images is first chopped into 16 (typically) sub-images for analysis (Fig. 5A), and how P and training set size vary relative to either camera images or sub-images (Fig. 5B).”.*

**2.16**: *Line #333 reference Fig. 6, while there are only 5 figures.*

**Response:**

We have modified the text to reflect the correct figure.Line 400. Now reads:

*“...are needed for junction reconstruction (Fig. 5).”.*
